# The Mann-Kendall-Sneyers test to identify the change points of COVID-19 time series in the United States

**DOI:** 10.1186/s12874-022-01714-6

**Published:** 2022-08-30

**Authors:** Xiang Chen, Hui Wang, Weixuan Lyu, Ran Xu

**Affiliations:** 1 Department of Geography, University of Connecticut, Storrs, CT 06269 USA; 2 Institute for Collaboration on Health, Intervention, and Policy (InCHIP), University of Connecticut, Storrs, CT 06269 USA; 3 Department of Geosciences, Mississippi State University, Mississippi State, MS 39762 USA; 4 Department of Allied Health Sciences, University of Connecticut, Storrs, CT 06269 USA

**Keywords:** Mann-Kendall-Sneyers, Epi curve, Time series, Nonparametric, COVID-19, Change point detection

## Abstract

**Background:**

One critical variable in the time series analysis is the change point, which is the point where an abrupt change occurs in chronologically ordered observations. Existing parametric models for change point detection, such as the linear regression model and the Bayesian model, require that observations are normally distributed and that the trend line cannot have extreme variability. To overcome the limitations of the parametric model, we apply a nonparametric method, the Mann-Kendall-Sneyers (MKS) test, to change point detection for the state-level COVID-19 case time series data of the United States in the early outbreak of the pandemic.

**Methods:**

The MKS test is implemented for change point detection. The forward sequence and the backward sequence are calculated based on the new weekly cases between March 22, 2020 and January 31, 2021 for each of the 50 states. Points of intersection between the two sequences falling within the 95% confidence intervals are identified as the change points. The results are compared with two other change point detection methods, the pruned exact linear time (PELT) method and the regression-based method. Also, an open-access tool by Microsoft Excel is developed to facilitate the model implementation.

**Results:**

By applying the MKS test to COVID-19 cases in the United States, we have identified that 30 states (60.0%) have at least one change point within the 95% confidence intervals. Of these states, 26 states have one change point, 4 states (i.e., LA, OH, VA, and WA) have two change points, and one state (GA) has three change points. Additionally, most downward changes appear in the Northeastern states (e.g., CT, MA, NJ, NY) at the first development stage (March 23 through May 31, 2020); most upward changes appear in the Western states (e.g., AZ, CA, CO, NM, WA, WY) and the Midwestern states (e.g., IL, IN, MI, MN, OH, WI) at the third development stage (November 19, 2020 through January 31, 2021).

**Conclusions:**

This study is among the first to explore the potential of the MKS test applied for change point detection of COVID-19 cases. The MKS test is characterized by several advantages, including high computational efficiency, easy implementation, the ability to identify the change of direction, and no assumption for data distribution. However, due to its conservative nature in change point detection and moderate agreement with other methods, we recommend using the MKS test primarily for initial pattern identification and data pruning, especially in large data. With modification, the method can be further applied to other health data, such as injuries, disabilities, and mortalities.

## Background

The Coronavirus Disease 2019 (COVID-19) pandemic has disrupted every aspect of human society. Because of the highly infectious nature of the disease, state governments in the United States (US) have implemented social distancing measures (e.g., closure of non-essential businesses, regional lock-down, and face-covering mandates) to contain the virus spread and flatten the epidemic curve (epi curve) [1]. However, since these state-level measures have differed in the strength and timeline of policy enforcement, it is intractable to rely on a simple rubric to evaluate the policy effectiveness. An alternative step is via analyzing the time series of the COVID-19 cases, which can eventually assist stakeholders with proactive health policymaking, such as determining the optimal timing to relieve social distancing.

One critical variable in the time series analysis is the change point, also called the inflection point, which is the point where a sudden change occurs in chronologically ordered observations. The change point detection has been long employed in statistical theory [2], but its applications to COVID-19 are relatively underexplored. For example, when modeling COVID-19 cases, the majority of studies have defined change points as key dates of policy interventions or social events [1, 3]. Other studies have employed parametric models, such as the linear regression model [4, 5] and the Bayesian model [6, 7] to derive change points. However, most of these parametric models require that the observations are normally distributed and that the trend line cannot have extreme variability. In situations where the observations show large variability over time and the trend line cannot be well fitted, parametric models become less reliable. These situations are not uncommon in fitting the COVID-19 epi curve, as the disease progression has a considerable degree of uncertainties and variability [1].

To overcome the limitations of the parametric model, we have applied a nonparametric model, called the Mann-Kendall-Sneyers (MKS) test, to change point detection in the COVID-19 epi curve. The MKS test, developed from a prototype model by Mann [8], is used to detect the monotonic trends (e.g., upward, downward) and their corresponding change points in time series data. The model has been primarily employed in earth science research to characterize the fluctuation of climatic and environmental variables, such as rainfall, air temperature, and surface runoff [9–11]. Recently, some COVID-19 studies have used the Mann-Kendall (MK) test, which is an earlier version of the MKS test, for trend detection [12, 13]. While the MK test is useful in detecting monotonic trends, it cannot detect changes in the trends and the corresponding change points, making it less useful for disease tracking and monitoring in the mid to long term. The MKS test, as a sequential extension of the MK test [14], fills this gap. It can become a valuable tool for long-term disease monitoring and can thus support public health decision-making.

The contributions of the paper are as follows.

- The paper is the first to apply the MKS test to COVID-19 time series analysis.
- The paper identifies six change point patterns for state COVID-19 cases.
- The paper develops an open-access tool for model implementation.

## Methods

The nonparametric MKS test [15], oftentimes called the sequential Mann-Kendall-Sneyers test, has been applied to the change point detection for long-term time series data (e.g., hydrological changes, climatic changes). According to the Centers for Disease Control and Prevention (CDC) report, both social distancing and mass gathering can potentially lead to an abrupt change in regional COVID-19 cases, albeit in different directions [16]. Then, we have evaluated the potential of the MKS test for change point detection in short-term time series data, the COVID-19 cases of infection.

In this section, we first articulate the MKS test. Then, we use an example to demonstrate the model implementation.

### Method description

The MKS test applied to the COVID-19 time series data can be completed in three major steps.

#### Step 1: Deriving test statistics (*S*_*k*_)

We have treated new weekly cases as an independent observation in a 45-week time series data. Under the null hypothesis that the development of new cases remains stable, for each state, we have a time series of the weekly new cases: *X* = {*x*_1_, *x*_2_, *x*_3_…*x*_*N*_ }, where *n* is the total number of weeks under observation (*N* = 45 in our case study). *m*_*i*_ (*i* = 1, 2, …, *N*) represents the total number of elements *x*_*j*_ preceding *x*_*i*_ (*j* < *i*) where *x*_*j*_ < *x*_*i*_.

Based on *m*_*i*_, the test statistic *S*_*k*_ derives the cumulative *m*_*i*_ for each week, as shown in Eq. (1).

1 $${S}_k=\sum_{i=1}^k{m}_i\ \left(k=1,2,3,\dots, N\right)$$

The mean of *S*_*k*_ can be derived by Eq. (2).

2 $$E\left({S}_k\right)=k\left(k-1\right)/4$$

The variance of *S*_*k*_ can be derived by Eq. (3).

3 $$VAR\left({S}_k\right)=k\left(k-1\right)\left(2k-5\right)/72$$

#### Step 2: Deriving two sequences (*U*_*f*_ and *U*_*b*_)

Next, we derive two sequences, the forward sequence *U*_*f*_ and the backward sequence *U*_*b*_, based on the three variables (*S*_*k*_, *E*(*S*_*k*_), and *VAR*(*S*_*k*_)) in Eqs. (1) through (3). Specifically, the forward sequence *U*_*f*_ of the time series is derived by Equation [4].

4 $${U}_f=\left({S}_k-E\left({S}_k\right)\right)/\sqrt{VAR\left({S}_k\right)}$$

Then, we reverse the sequence of the original time series *X* and term it *X*_*r*_. An intermediate sequence *U*_*fr*_ is derived by applying Eq. (4) to the reversed time series *X*_*r*_. We reverse the sequence of the values in *U*_*fr*_ (i.e., the first value appears the last, and vice versa). We generate the backward sequence *U*_*b*_ by adding a negative sign to the reversed values.

#### Step 3: Deriving change points

Lastly, we identify the change points of the time series *X* based on the two generated sequences (*U*_*f*_ and *U*_*b*_). We first identify the initial set of the change points as the points of intersection between the two sequences. Previous studies show that it is uncertain to recognize all of these change points as abrupt changes, as a change point can be induced by a sudden shift of the mean value over two stable periods [17]. These outlier points could be reevaluated by using additional detection methods, such as the double mass curve [18]. To avoid miscounting the change points while making the proposed method more applicable, we employ a statistical filter—the points of intersection falling beyond the 95% confidence intervals (CIs), which correspond to Z-scores = ±1.96, are rejected. This filter has been used in relevant MKS studies [19]. It is worth noting that the MKS test can also identify the monotonic trend or the change of direction—if a point of intersection is between the Z-scores of 0 and 1.96, the change is upward; if the point is between the Z-scores of − 1.96 and 0, the change is downward.

### Model implementation

In this section, we take the state of Virginia as an example to further elaborate on the model implementation. The MKS test can be implemented in Microsoft Excel by calling embedded functions. The datasets and codes are available on GitHub (https://github.com/peterbest52/mks).

#### Data cleaning

Daily confirmed cumulative COVID-19 case data between March 22, 2020 and January 31, 2021 (in a total of 45 weeks) were obtained from the USAFacts website (https://usafacts.org/data/). Then, we aggregated the data on a weekly basis, generating a 45-week time series for each state representing new weekly cases. Lastly, to demonstrate the method, we extracted the data for Virginia as the time series *X*.

#### MKS test

**Fig. 1:**
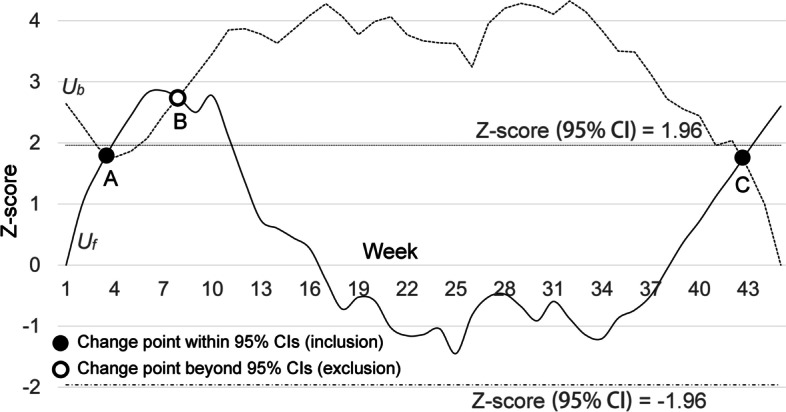
MKS test of new weekly cases in Virginia with the forward sequence (solid line) and the backward sequence (dashed line). The black dot is the identified change point, and the white dot is the excluded change point

For time series *X*, we derived *m*_*i*_, the cumulative times that the case value of the current week is larger than that of each preceding week. Following this step, *S*_*k*_ was derived as the cumulative *m*_*i*_ (*i* = 1, 2, …, *k*), according to Eq. (1); then, the mean value of *S*_*k*_ or *E*(*S*_*k*_) and the variance of *S*_*k*_ or *VAR*(*S*_*k*_) were derived by Eqs. (2) and (3), respectively. It is worth noting that, since *k* is the only independent variable in Eqs. (2) and (3), *E*(*S*_*k*_) and *VAR*(*S*_*k*_) are the same for all states in this study. Based on Eq. (4), we derived the forward sequence *U*_*f*_ for Virginia (solid line in Fig. 1).

Then, we reversed the time series *X* and derived *X*_*r*_. We derived the intermediate sequence *U*_*fr*_ by applying Eq. (4) to *X*_*r*_. Lastly, we derived the backward sequence *U*_*b*_ (dashed line in Fig. 1) by first reversing the sequence of values in *U*_*fr*_ and then adding a negative sign to these values.

#### Change point detection

The forward sequence (*U*_*f*_) and the backward sequence (*U*_*b*_) were plotted as the solid line and dashed line, respectively (Fig. 1). The points of intersection between the two sequences became the initial set of the change points. The thresholds of 95% CIs (Z-scores = ± 1.96) were set as the statistical filter. Only change points within the thresholds were retained. Specifically, in the case of Virginia, three points of intersection were initially detected. Week 4 (Point A in Fig. 1) and Week 43 (Point C in Fig. 1) were identified as the final change points with statistical confidence. Week 8 (Point B in Fig. 1) was excluded (Z-score = 2.72), as it fell beyond the thresholds. Since both Point A and Point C were between Z-scores of 0 and 1.96, these changes were upward.

## Results

By applying the MKS test to weekly new COVID-19 cases in 50 states, we identified that 30 states (60.0%) have at least one change point within the 95% CIs. For the unqualified states, most of them have no change points within the 95% CIs but have at least one change point beyond the 95% CIs. Only the state of Vermont has no change points either within the 95% CIs or beyond, meaning that there is no abrupt case decrease or increase during the entire study period.

**Fig. 2:**
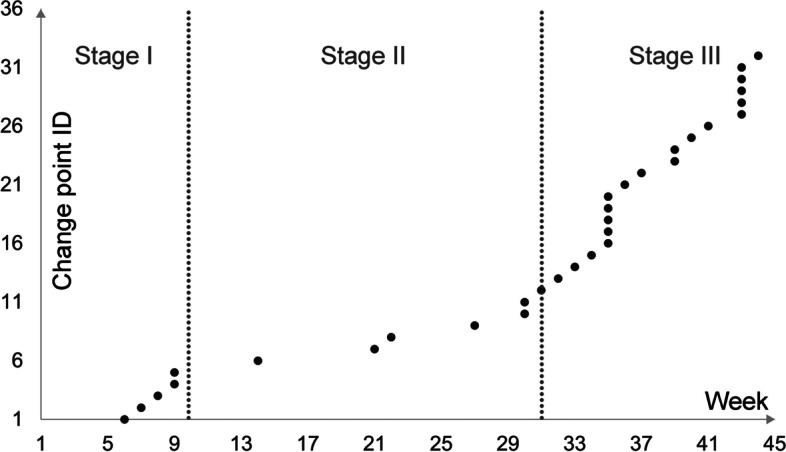
The three development stages based on clusters of chronologically ordered change points

**Fig. 3:**
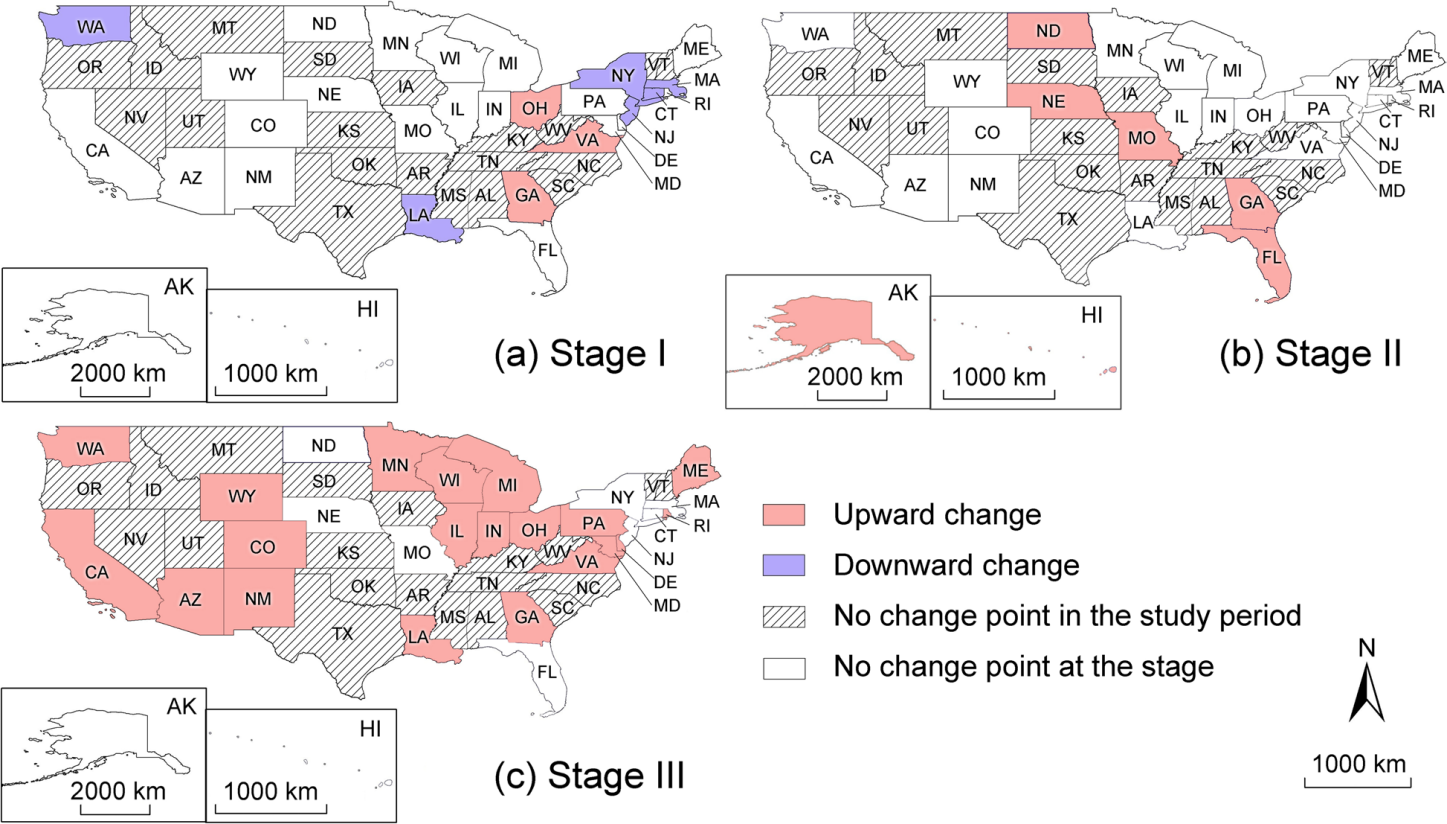
The emergence of the change point for each state **a** at the first stage (Weeks 1–10), **b** at the second stage (Weeks 11–30), and **c** at the third stage (Weeks 31–45). The map is created by the authors

To characterize the temporal distribution of these change points, we further divided the study period into three disease development stages, namely, Weeks 1–10 (March 23 through May 31, 2020), Weeks 11–30 (June 1 through November 19, 2020), and Weeks 31–45 (November 19, 2020 through January 31, 2021). These three stages were determined by the three clusters of chronologically ordered change points, as shown in Fig. 2. Based on the three development stages, we then mapped out the emergence of the change point for each state, as shown in Fig. 3.

**Fig. 4:**
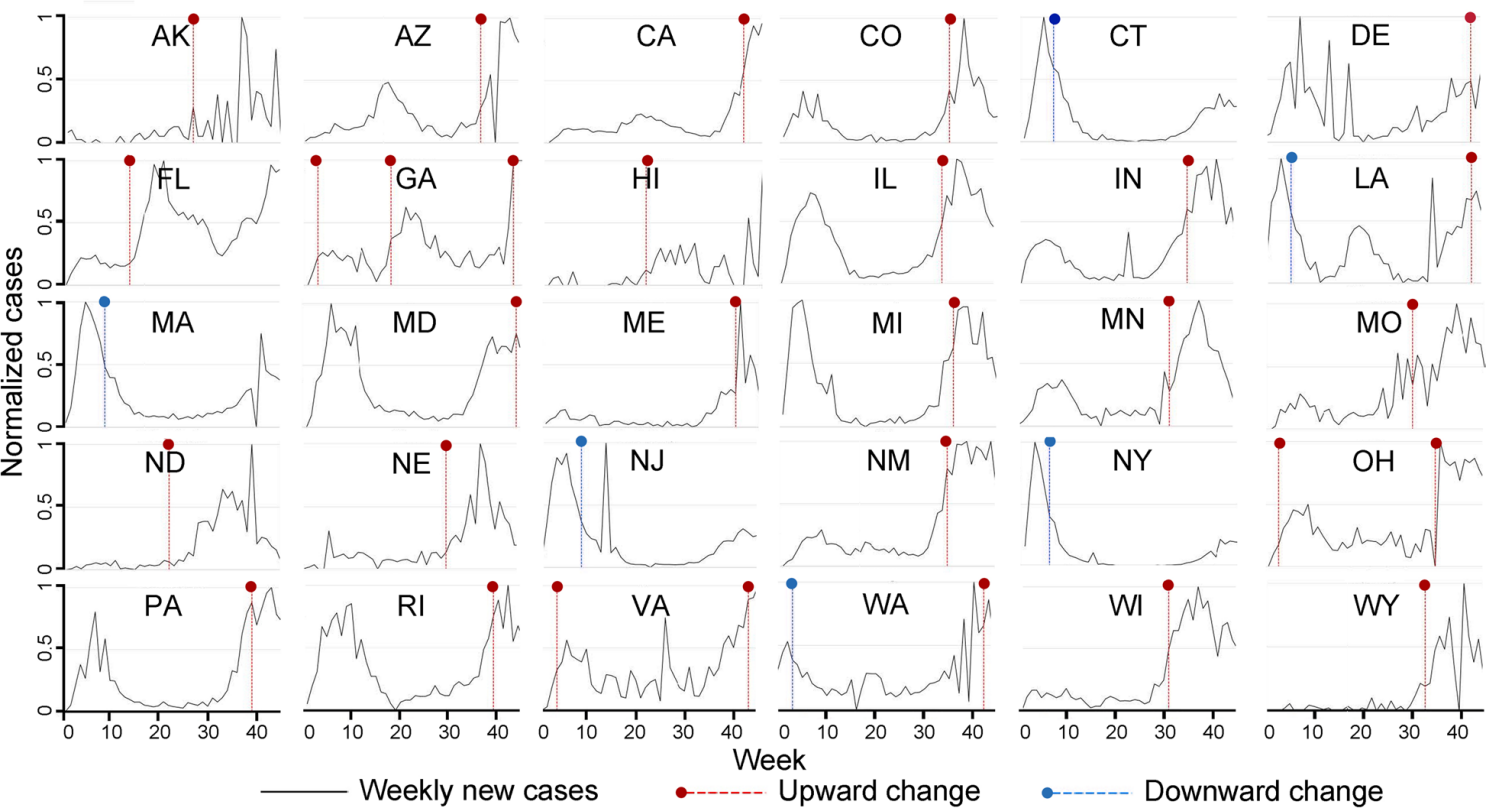
States with at least one change point identified. The horizontal axis is the week; the vertical axis is the weekly new cases normalized to 0–100% with respect to the maximum weekly new cases in each state

Figure 4 shows the change points detected by the MKS test for the 30 states with at least one change point within the 95% CIs. Among these states, we identified that a single change point exists for 25 states, two change points exist for 4 states (i.e., LA, OH, VA, and WA), and three change points exist for one state (i.e., GA). Then, we further derived 6 change patterns based on the emergence and direction of the change point at the three stages, as shown in Table 1.

**Table 1:** Summary of change patterns based on the emergence and direction of change points at three stages

No.	Pattern	State
1	+++	GA
2	+/+	OH, VA
3	−/+	LA, WA
4	−//	CT, MA, NJ, NY
5	/+/	AK, FL, HI, MO, ND, NE
6	//+	AZ, CA, CO, DE, IL, IN, MD, ME, MI, MN, NM, PA, RI, WI, WY

+ upward change point, − downward change point, / no change point

## Discussion

Two epidemiologic patterns can be identified in Table 1. First, the downward changes at the first stage (Pattern 4) appear only in Northeastern states (e.g., CT, MA, NJ, NY), as confirmed in Fig. 3a. This pattern can be explained by the immediate state policy actions on social distancing in this region during the early outbreak. After COVID-19 was declared a national emergency by the presidential proclamation on March 1, 2020 [20], most Northeastern states enforced social distancing regulations in late March and early April, including the closure of non-essential businesses and schools [21]. These policies largely restricted face-to-face interactions, slowed the virus diffusion, and eventually, suppressed the epi curves. Second, the upward changes at the third stage appear mostly in the Western states (e.g., AZ, CA, CO, NM, WA, WY) and the Midwestern states (e.g., IL, IN, MI, MN, OH, WI), as shown in Fig. 3c. This result is consistent with the observation that most Western and Midwestern states experienced an abrupt case surge in the late summer and fall [22]. The rising trend could be linked to their less restrictive reopening policies, especially reopening indoor dining without a statewide face-covering mandate [23].

To further validate the MKS test, we compared it with two other change point detection methods, the pruned exact linear time (PELT) method and the regression-based method (Table 2), both of which are commonly used for detecting multiple change points in time series data. Specifically, the PELT method searches for change points by minimizing a cost function over possible numbers and locations of change points, and it implements an efficient pruning to increase the computational efficiency [24, 25]. The regression-based method analyzes the time series using a regression model with multiple segments, where the coefficients shift from one stable regression relationship to another. It implements a dynamic programming approach to find segments that can minimize the residual sum of squares [26, 27]. We implemented the PELT method using the ‘changepoint’ package in R [25] and the regression-based method using the ‘strucchange’ package in R [28].

**Table 2:** Summary of the identified change points (CP) by the three methods

	MKS			PELT		Regression-based			
State	CP#1	CP#2	CP#3	CP#1	CP#2	CP#1	CP#2	CP#3	CP#4
AK	27			36		36			
AL				41		39			
AR				36		18	25	37	
AZ	37			40		38			
CA	41			40		39			
CO	36			34		10	34		
CT	8			9		10	35		
DE	43			14	38	14	38		
FL	15			15		16	22	29	39
GA	3	18	43	42		17	24	39	
HI	21			44		22			
IA				34		34			
ID				31		17	33	39	
IL	34			33		12	33		
IN	35			34		34			
KS				36		27	36		
KY				36		30	38		
LA	6	44		7	34	7	34		
MA	9			11		11	37		
MD	44			12	36	6	12	35	
ME	40			36		36			
MI	36			7	33	7	33		
MN	32			33		33	39		
MO	30			26		26	35		
MS				42		15	37		
MT				30		18	30		
NC				40		36			
ND	23			27		27	33	39	
NE	30			33		33	39		
NH				40		14	36		
NJ	9			14		14			
NM	35			34		34			
NV				36		17	24	36	
NY	6			7		7			
OH	3	36		35		12	35		
OK				36		17	31	39	
OR				35		35			
PA	39			36		11	36		
RI	39			13	36	13	36		
SC				40		16	23	39	
SD				31		26	32	38	
TN				36		17	31	37	
TX				39		17	23	33	39
UT				33		33			
VA	4	43		37		37			
VT				36		6	36		
WA	4	43		40		7	38		
WI	31			30		30	39		
WV				36		31	37		
WY	33			34		34			

The validation tested if the MKS-identified change points can be confirmed by the two other methods. A confirmation is accepted if an MKS-identified change point is validated by another method within a two-week window. The comparison results are shown in Table 2. Based on the 36 MKS-identified change points, the MKS-test reaches 41.7% agreement (15/36) with the PELT method and 47.2% agreement (17/36) with the regression-based method. It is also worth mentioning that the other two methods identified at least one change point for every state, even when there is no obvious change of direction. The comparison results signify that the MKS test is a relatively conservative method for change point detection, as it can only detect abrupt changes and can thus avoid false-positive results.

## Conclusions

To sum up, the MKS test has several advantages in change point detection. First and foremost, it is characterized by high computational efficiency and easy implementation. Users can easily implement this method in Microsoft Excel without any prior statistical knowledge or modeling skills. Second, the method can detect the change of direction, whereas some other methods (e.g., PELT) can only identify the existence of a change without specifying the direction. Third, since the MKS test is a nonparametric model, it can be applied to time series data where the distribution is not normal or has extreme variability. However, due to its conservative nature and moderate agreement with the other slower but more sensitive methods, we recommend using the MKS test primarily for initial pattern identification and data pruning, especially in large data. For example, to identify the change points in a long sequence of COVID-19 infection data, we can first use the MKS test to narrow down the time window where changes are likely to occur, and then use a second method (which has a higher computational cost but is more sensitive) to reconfirm the change pattern. In addition, as the conservativeness of the MKS test can be easily modified by adjusting the width of the statistical filter, future studies should examine how the quality of the results derived from the MKS test may vary as a function of the statistical filter.

This pilot study is the first to implement the MKS test for COVID-19 studies. An open-access tool is developed to facilitate the model implementation. With further validation and modification, the method can be applied to other health data, such as injuries, disabilities, and mortalities. By identifying key time points where chronologically ordered observations have a drastic change, the method can eventually contribute to revealing the etiology of these health outcomes and supporting public health decision-making.

## Data Availability

The data and codes for the study can be accessed on Github [https://github.com/peterbest52/mks].

## Abbreviations

CDC: Centers for Disease Control and Prevention
CI: Confidence interval
COVID-19: Coronavirus Disease 2019
CP: Change point
MKS: Mann-Kendall-Sneyers
PELT: Pruned exact linear time
US: United States
